# An Extremely Rare, Remote Intracerebral Metastasis of Oral Cavity Cancer: A Case Report

**DOI:** 10.1155/2013/257046

**Published:** 2013-10-07

**Authors:** Mario Leimert, Tareq A. Juratli, Claudia Lindner, Kathrin D. Geiger, Johannes Gerber, Gabriele Schackert, Matthias Kirsch

**Affiliations:** ^1^Department of Neurosurgery, University Hospital Carl Gustav Carus, Technical University of Dresden, Fetscherstraße 74, 01307 Dresden, Germany; ^2^Department of Neuropathology, Technical University of Dresden, Fetscherstraße 74, 01307 Dresden, Germany; ^3^Department of Neuroradiology, Technical University of Dresden, Fetscherstraße 74, 01307 Dresden, Germany

## Abstract

Distant brain metastases from oral squamous cell carcinomas (OSCC) are extremely rare. Here we describe a case of a 53-year-old man with a primary OSCC who referred to the neurosurgical department because of epileptic seizures. MR imaging revealed an enhancing lesion in the right parietal lobe. A craniotomy with tumor removing was performed. Histopathological examination verified an invasive, minimally differentiated metastasis of the primary OSCC. The patient refused whole brain radiation therapy and died from pulmonary metastatic disease 10 months after the neurosurgical intervention without any cerebral recurrence. To the authors' knowledge, only two similar cases have been previously reported.

## 1. Introduction

Remote brain metastases from oral squamous cell carcinomas (OSCC) are an extremely rare occurrence. To date, only few cases have been reported previously [1, 2]. In contrast, direct intracranial invasion is not infrequent in patients with nasopharyngeal carcinoma (NPC) at a locally advanced stage [3]. Incidence of brain metastases following NPC may be increasing secondary to advancements in the treatment of systemic disease and earlier detection by more sensitive imaging modalities [4]. Most distant metastases from squamous cell carcinoma (SCC) are reported to occur in the liver, lungs, and bones [5]. Therefore, preoperative tumor staging is focussed on these sites (CT scan of the chest, radionuclide bone scans, and ultrasound of the liver). In the following case study, we present a patient who developed a histologically confirmed brain metastasis of OSCC. The patient developed symptoms from his cerebral metastasis 29 months after the primary disease was diagnosed.

## 2. Case Description

A 53-year-old man with a 29-month history of a slowly enlarging ulcer on the bottom of the right lateral oral cavity was referred to our Department of Head and Neck surgery.

After biopsy, a radical surgical resection of the tumor with supraomohyoid and functional neck dissection in continuity and reconstruction with a radial forearm free flap was performed. Histopathological work-up diagnosed a primary oral squamous cell carcinoma stage T3N3 (Figure 3(a)). Based on the stage of this diagnosis, adjuvant radiotherapy was initiated with a total dose of 64 Gy delivered in 32 fractions to both sides of the neck and the primary site. A CT scan revealed bilateral small pulmonary nodules, which were diagnosed as pulmonary metastases, but the patient declined chemotherapy. After radiation therapy, he was well and with stable disease for 26 months. Then, after a 3-week period of general weakness, he developed epileptic seizures which initiated further diagnostic work-up.

**Figure 1:**
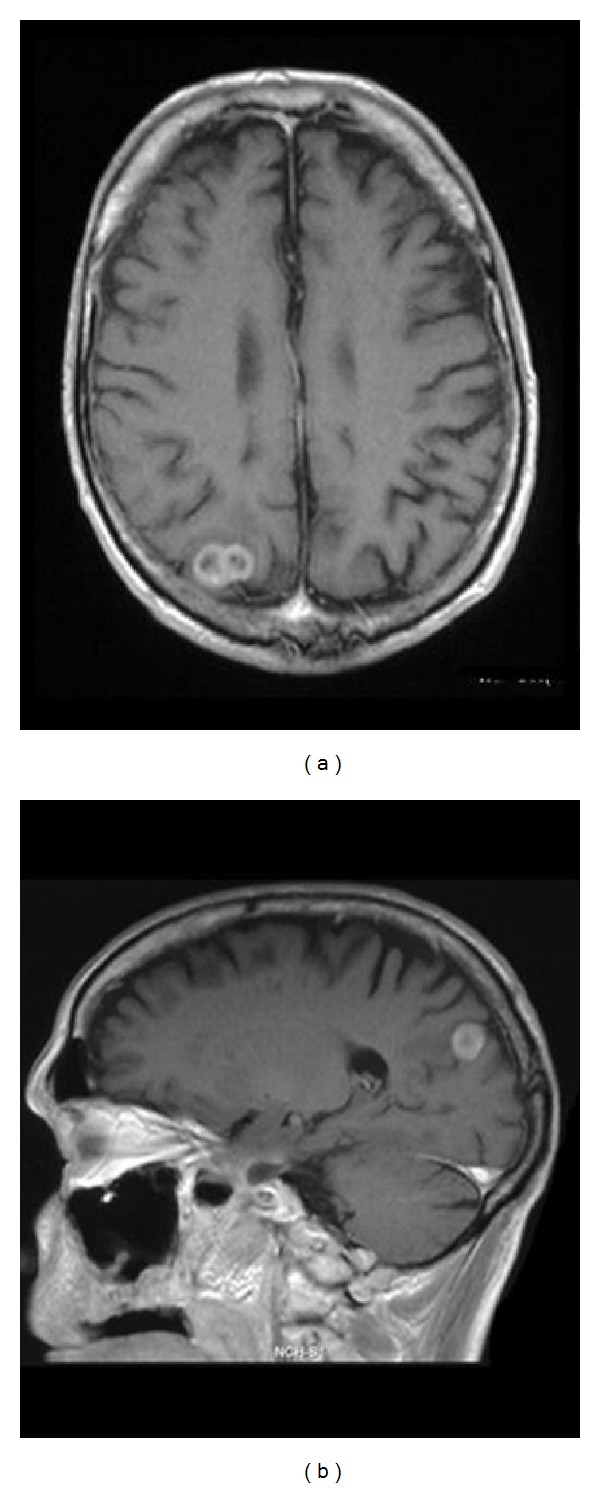
Axial (a) and sagittal (b) magnetic resonance scans (T1w with Gd) reveal an enhancing lesion with central necrosis in the right parietal lobe which is typically located in the cortical/subcortical layer.

**Figure 2:**
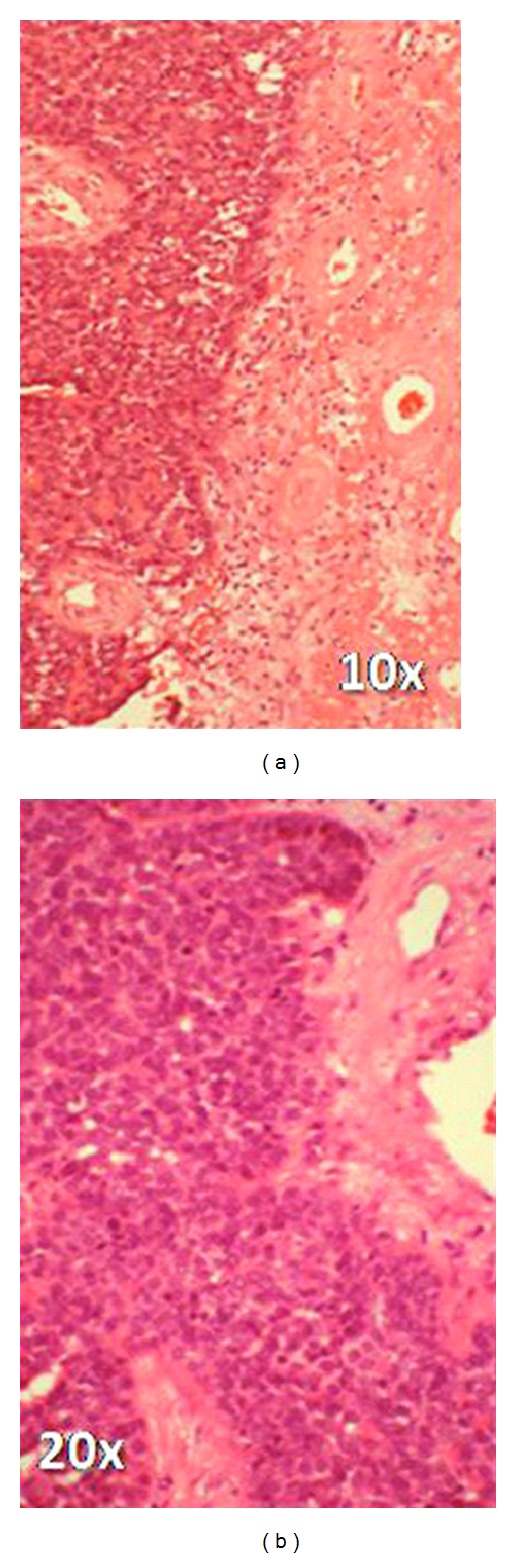
Histological diagnosis of the cerebral metastasis revealed a minimally differentiated squamous cell carcinoma with single horn pearls, solid growth, pleomorphic nuclei, and numerous mitosis, adjacent to edematous brain tissue with extensive reactive gliosis. H and E stain.

**Figure 3:**
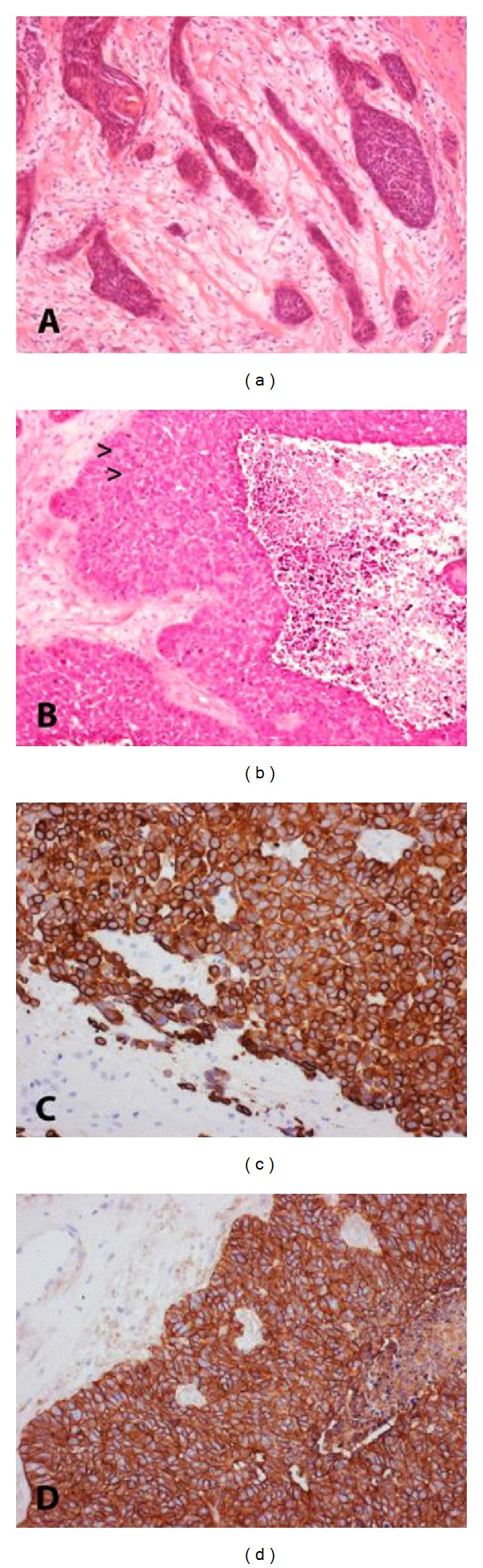
Histology and immunohistochemistry of OSCC, 5 *μ*m thick serial sections of both primary tumor and cerebral metastasis were stained with H&E. Immunochemistry was performed using an indirect peroxidase system with nonbiotinylated polymer secondary antibodies following the instructions of the manufacturer (MEDAC). Diaminobenzidine (Sigma, brown) is used as a chromogen. Magnification: original × 20. (a) Primary intermediately differentiated squamous cell carcinoma of the oral cavity with recognizable squamous cell differentiation. (b) Cerebral metastasis of poorly differentiated squamous cell carcinoma containing few horn pearls (arrow heads) and central necrosis. (c) Cerebral metastasis of OSCC, immunocytochemistry for CK 5/6, a cytokeratin marker indicative for squamous cell carcinoma with completely positive brown reaction product on the plasma membrane of nearly all tumor cells. Adjacent brain tissue shows gliosis but remains negative for CK-5/6 (light blue). (d) Cerebral metastasis of OSCC, immunohistochemistry for EGFR shows strong overexpression with complete staining of the cell membranes in all vital tumor cells. Note the negative results in remaining brain parenchyma (light blue).

MR imaging revealed a heterogeneously enhancing lesion of approximately 2 × 1 cm in the right parietal lobe, typically located in the cortical/subcortical area (Figure 1). The patient was now assessed as T3N3M1, and surgical resection of the suspected brain metastasis was advised. Preoperative computed tomography (CT) of the chest showed progress of the pulmonary metastases. A craniotomy was performed, and the tumor was completely removed judged upon intraoperative microscopic and postoperative MR imaging. Histopathological examination verified an invasive, minimally differentiated metastasis of the primary OSCC (Figures 2 and 3(b)–3(d)). The patient refused whole brain radiation therapy (30 Gy) and died from pulmonary metastatic disease 10 months after the neurosurgical intervention without any cerebral recurrence.

## 3. Discussion

Malignant tumors can metastasize into every part of the brain. The underlying factors relating to the differential propensity of primary tumors to metastasize into the brain remain unknown. OSCC classically metastasizes to cervical lymph nodes first. The mechanism of spread is probably lymphatic. Late presentation of distant metastatic disease from OSCC is increasingly reported, in particular those metastases that are most likely the cause of death after locoregional control of the primary tumor was achieved [6]. It has been suggested that late-occurring metastases may result from differences in the proliferative potential of a subgroup of cells in the growth-arrested metastatic tumor [7]. Overexpression of epidermal growth factor receptor (EGFR), as in our case (Figure 3(d)), has been found in various epithelial malignancies, including head and neck squamous cell carcinoma (HNSCC), and is associated with increased tumor growth, metastasis, resistance to chemotherapeutic agents, and poor prognosis [8]. Treatment with EGFR inhibitors with chemotherapy can significantly down regulate EGFR expression and inhibit cell growth of HNSCC. Therefore, EGFR inhibitors are routinely used in the treatment of HNSCC. Moreover, a recently published retrospective study with seven HNSCC patients who developed cerebral metastases, demonstrated a correlation with human papillomavirus (HPV) in four out of seven cases [2]. The authors concluded in their work that, due to the potential for developing brain metastases from HPV-related HNSCC long after curative therapy, an extended follow-up is needed in this patients' collective.

In patients with systemic cancer, parenchymal or leptomeningeal metastases can cause epilepsy, and potentially reversible medical and neurologic deficits can lead to acute symptomatic seizures [9]. Surgical removal of symptomatic lesion followed by external adjuvant radiotherapy is considered the best treatment in patients with single brain metastases and with systemic disease which is under control or absent [10].

## 4. Conclusion

Our understanding of the complex interactions between metastatic tumor cells and the host environment should improve. With development in systemic therapy, the incidence of central nervous system metastases has increased in patients with cancer [11]. The described case confirmed the previously reported occurrence of cerebral metastases of OSCC. Both patients have had their primary disease for longer than 12 months. According to this knowledge, a brain metastasis should be taken in consideration in case of neurological deterioration. Furthermore, we could detect the known EGFR within a brain metastasis as a critical protein for survival of epithelial tissues.
